# Pembrolizumab combined with anlotinib improves therapeutic efficacy in pulmonary sarcomatoid carcinoma with TMB-H and PD-L1 expression: a case report and literature review

**DOI:** 10.3389/fimmu.2023.1274937

**Published:** 2023-10-23

**Authors:** Shugui Wu, Shanlian Wu, Xiaohong Liao, Chaoming Zhou, Feng Qiu, Chen Wang, Wenjuan Zhong

**Affiliations:** ^1^ Department of Oncology, The Affiliated Ganzhou Hospital of Nanchang University, Ganzhou, China; ^2^ Department of Oncology, Ganzhou Hospital-Nanfang Hospital, Southern Medical University, Ganzhou, China; ^3^ Department of Pathology, Ganzhou Hospital-Nanfang Hospital, Southern Medical University, Ganzhou, China; ^4^ Department of Oncology, The First Affiliated Hospital of Nanchang University, Nanchang, China; ^5^ Department of Oncology, The First Affiliated Hospital of Gannan Medical University, Ganzhou, China

**Keywords:** pulmonary sarcomatoid carcinoma, pembrolizumab, anlotinib, tumor mutation burden, case report

## Abstract

**Background:**

Pulmonary sarcomatoid carcinoma (PSC) is a unique subtype of non-small cell lung cancer (NSCLC) with a high degree of malignancy and poor therapeutic effects. With the widespread use of immune checkpoint inhibitors (ICIs) in recent years, few studies have reported that immunotherapy is effective against PSC. As a multi-target anti-vascular targeting agent, anlotinib showed a better anti-tumor effect in various cancer species. The paper reported the therapeutic and side effects of pembrolizumab combined with anlotinib in a patient with advanced PSC.

**Case presentation:**

This is a 73 year old female patient who underwent thoracoscopy right upper lobectomy and was diagnosed as locally advanced PSC. However, the patient experienced tumor recurrence and metastasis 7 weeks after surgery and was unable to tolerate chemoradiotherapy. Moreover, she detected TP53 mutation and found that tumor mutation burden (TMB) and PD-L1 were high expression. Therefore, the patient received pembrolizumab combined with anlotinib treatment. After 15 cycles of treatment, the tumor significantly shrank with no tumor activity. The evaluation of tumor efficacy is partial response (PR). During the treatment period, she experienced one-degree thyroid-stimulating hormone elevation and two-degree hand-foot syndrome. Pembrolizumab and anlotinib was continued for two years as a maintenance treatment. The patient had a good quality of life and no disease progression was observed. Currently, the patient is still alive without tumor progression and has overall survival exceeding 45 months and toxic side effects were tolerable.

**Conclusions:**

Combining ICIs and anti-angiogenic targeted therapy has brought new hope in treating advanced PSC. Additionally, TMB and PD-L1 expression could be potential predictive biomarkers of the efficacy in advanced PSC with immunotherapy.

## Introduction

PSC is a rare histological subtype of NSCLC, accounting for 0.5% of the total incidence rate of NSCLC (1). It is common in middle-aged and elderly men (73%), and 94.6% of patients are attributable to smoking (2). PSC has characteristics of both epithelial and mesenchymal tumors (3). According to the 2015 World Health Organization (WHO) classification of lung tumors, PSC can be divided into five subtypes: pleomorphic carcinoma, spindle cell carcinoma, giant cell carcinoma, carcinosarcoma, and pulmonary blastoma (4). PSC patients present with more advanced stages and worse survival outcomes than other NSCLC patients, according to the SEER database (5).

PSC is clinically challenging mainly due to its low incidence rate, rare pathological type, rapid growth, easy distant metastasis, poor systemic treatment effect and difficulty in early diagnosis. The treatment of PSC largely follows the principles of NSCLC. Surgical resection is the standard treatment for early-stage patients, while more than 70% of PSC patients have locally advanced or distant metastasis at the time of diagnosis, losing the opportunity for radical surgery (1, 5). Even after early radical surgery, recurrence and metastasis are prone to occur (6). Postoperative adjuvant radiotherapy does not prolong patients’ overall survival (OS) with PSC (7). Chemotherapy is the primary treatment for advanced PSC patients but has low sensitivity. The objective response rate (ORR) of platinum-based first-line chemotherapy is only 8% (8). The median OS of patients with advanced PSC is only 5.0 months, and the five-year OS rate is 8.5% (5).

Researchers have found multiple gene mutations in PSC. Among them, TP53 and KRAS mutations are the most common genomic changes in the sarcomatoid carcinoma group, up to 74% and 34% (9). In addition, recent multi-omics analysis revealed high PD-L1 expression or high TMB of PSC (10–12). More than 60% of pure PSC patients (38/58) in China have MSI-H, PD-L1 positive or TMB high tumors (11). The study suggested the efficacy of ICIs in PSC: the ORR was 40.5%, and DCR was 64.8%, regardless of PD-L1 status. Median OS was 12.7 months (2). ICIs may serve as a new potential therapeutic option. Pembrolizumab is a humanized monoclonal anti-PD-1 antibody widely studied in many malignant tumors. Anlotinib is a multi-target tyrosine kinase inhibitor with broad-spectrum inhibitory effects on tumor angiogenesis and growth (13). Antiangiogenic agents have the potential to reprogram the immunosuppressive tumor microenvironment and prompt tumor vessel normalization. Furthermore, anlotinib can inhibit the expression of PD-L1 on vascular endothelial cells, thereby breaking through the “immune tolerance barrier” and promoting CD8+T cell infiltration to improve the balance of CD8/FoxP3 (14). Besides, TP53 mutations may represent a biomarker for predicting salutary effects of anlotinib in NSCLC (15). The combination of ICIs and anti-angiogenic inhibitors shows synergistic anti-tumor effects in various cancers (16–19). The advanced NSCLC patients received the first-line sintilumab combined with anlotinib and had a high objective response rate (ORR, 72.7%) and disease control rate (DCR, 100%) (16). However, there is no clinical study of ICIs in conjunction with anti-angiogenic drugs in PD-L1 positive PSC. Herein, we reported the efficacy and adverse effects of pembrolizumab combined with anlotinib in advanced PSC patient with high PD-L1 expression, TMB-H and TP53 mutation. In addition, potential biomarkers for effective treatment of advanced PSC were explored.

## Case presentation

**Figure 1:**
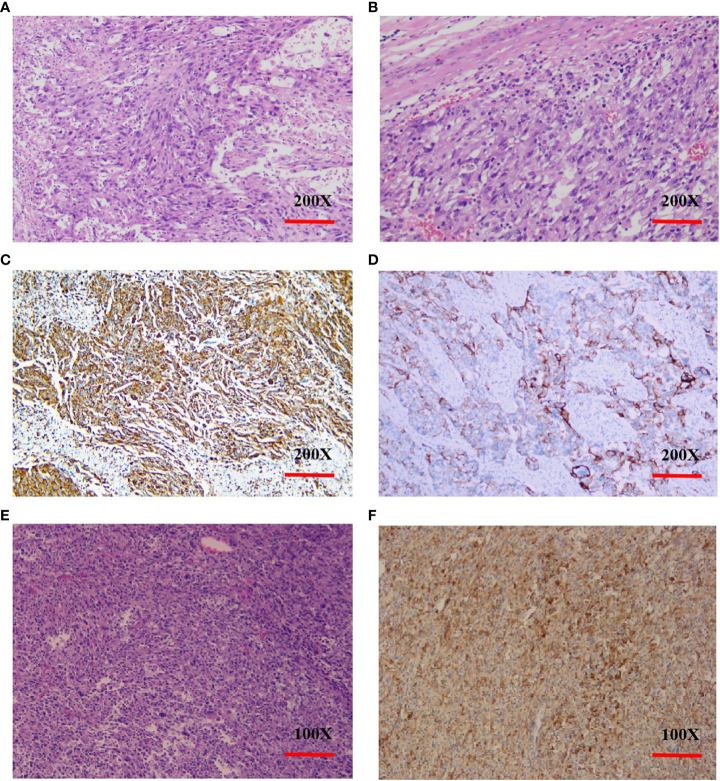
Histopathology and immunohistochemistry (IHC) of PSC. **(A, B)**, H&E stain, original magnification ×200. **(C)**, IHC Vimentin(+++), original magnification ×200. **(D)**, IHC CK(+), original magnification ×200. **(E)**, H&E stain. original magnification ×100. **(F)**, PD-L1 IHC (antibody 22C3 pharmDx), original magnification ×100. Tumor Proportion Score [^a^TPS]: 80% and Combined Positive Score [^b^CPS]: 95. (a.TPS was defined as the percentage of viable tumor cells with partial or complete membrane staining of PD-L1 in at least 100 viable tumor cells. b.CPS was defined as the number of PD-L1 stained cells (tumor cells, lymphocytes, macrophages) divided by the number of all tumor cells and multiplied by 100.).

**Figure 2:**
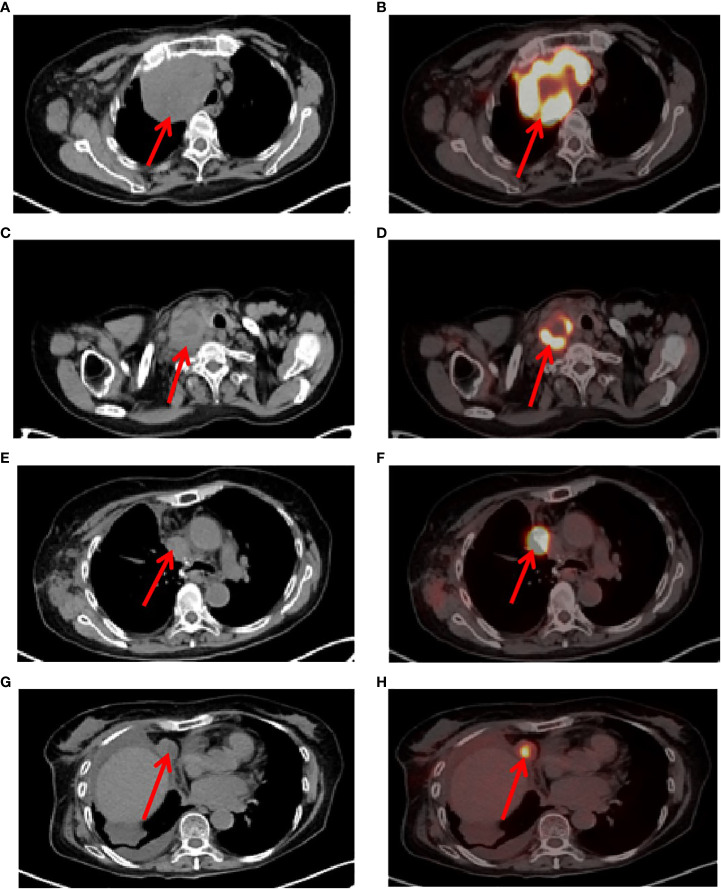
Tumor recurrence and metastasis region. **(A, B)**, 7 weeks after surgery, positron emission tomography/computed tomography (PET-CT) revealed tumor recurrence and metastasis, involving a huge mass shadow in the right-side area of the mediastinum (solid tumor, dmax = 70 mm * 44 mm), abnormal increase in FDG metabolism, SUVmax 28, **(C, D)**, Supraclavicular fossa lymph nodes, **(E, F)**, right mediastinum lymph nodes, **(G, H)**, right cardio-diaphragmatic angle metastatic lymph nodes.

**Figure 3:**
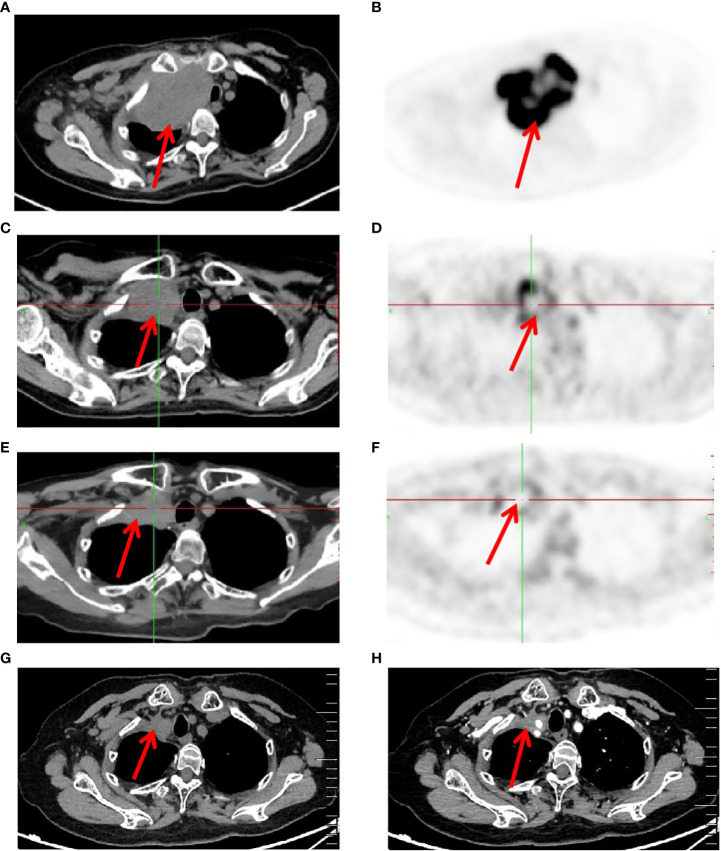
Imaging changes of patients before and after treatment. **(A, B)**, Baseline PET-CT findings of the patient’s course (solid tumor, dmax = 70 mm * 44 mm, abnormal increase in FDG metabolism and SUVmax 28). **(C, D)**, After 7 cycles of treatment (solid tumor, dmax = 54 mm * 43 mm, abnormal increase in FDG metabolism and SUVmax 7.4). **(E, F)**, After 15 cycles of treatment (solid tumor, dmax = 46mm * 33mm, and no tumor activity and SUVmax 2.4). **(G, H)**, After 34 cycles of treatment Computer Tomography results (solid tumor, dmax = 22mm * 20mm).

A 73-year-old female patient was admitted to the hospital on November 6, 2019 with a one-month history of cough and chest tightness. The patient had no history of smoking, drinking or family history of tumors. Following the admission, the evaluation and examination were conducted. 18F-fluorodeoxyglucose positron emission tomography/computed tomograph(18F-FDG PET/CT)showed a soft tissue mass shadow near the hilum of the right upper lobe tip, having a size of 62mm*49mm, and enlarged lymph nodes in the mediastinum (**Supplementary Material 1**). Moreover, the neuron specific enolase (NSE) biomarker level was elevated at 32.34 ng/ml (normal levels, <16.3 ng/ml). It was diagnosed as NSCLC. Then, she received the right upper lobe resection and lymph node dissection were performed under thoracoscopy in November 12, 2019, postoperative pathology revealed a right upper lung spindle cell malignant tumor, conformed to sarcoma carcinoma (**Figures 1A, B**), with low differentiation and a maximum diameter of about 8.5 cm. The tumor penetrated the dirty layer, parietal pleura, and accumulated in the hilar soft tissue with large tumor necrosis, vascular thrombus, nerve invasion, and hilar lymph node cancer metastasis, and negative surgical margin. A total of 43 mediastinal lymph nodes were dissected, of which 8 showed metastasis. Immunohistochemistry (IHC) showed Vim(+++, **Figure 1C**), CK(+, **Figure 1D**), CD34(+), CD68(+), Ki-67(60%+), P40 (–), P63(-), CK7(+), NapsinA(-), ALK(D5F3)(+), TTF-1(-), S-100(-), BRG1(+). The postoperative diagnosis was PSC IIIB stage (pT4N2M0, 8th edition AJCC). Seven weeks after surgery, PET-CT revealed tumor recurrence and metastasis (**Figure 2**), involving a huge mass shadow in the right-side area of the mediastinum (70 mm * 44 mm), abnormal increase in FDG metabolism, SUVmax 28 (**Figures 3A, B**), and right upper and lower clavicle socket, right axilla, mediastinum, and right cardio-diaphragmatic angle showed metastatic lymph nodes. Considering the tumor’s rapid progression, the patient could not withstand high-intensity chemoradiotherapy. Additionally, she received the next generation sequencing (NGS) of tumor tissue samples to detect four types of 1021 genes related to tumor occurrence and development: including point mutation, small fragment insertion and deletion, copy number variation and currently known fusion gene variation. NGS was conducted on the Gene+Seq-200/2000 platform of the College of American Pathologists (CAP) accredited clinical laboratory (Gen+, BeiJing, China). NGS and IHC results are presented in (**Supplementary Material 2**). The patient had high TMB (TMB-H, 11.52Muts/Mb), microsatellite stabilization (MSS), and PD-L1 was strongly expressed, Tumor Proportion Score [TPS]: 80% and Combined Positive Score [CPS]: 95 (**Figures 1E, F**). The profile of genetic alterations included TP53 mutation, with an abundance of 25.3% (**Supplementary Material 2**). Consequently, with the patient’s consent, pembrolizumab combined with anlotinib began treatment in January 2020. She was administered 200 mg pembrolizumab intravenously over 30 min every 3 weeks and received anlotinib orally at 10 mg day 1-14 every 3 weeks.

**Figure 4:**
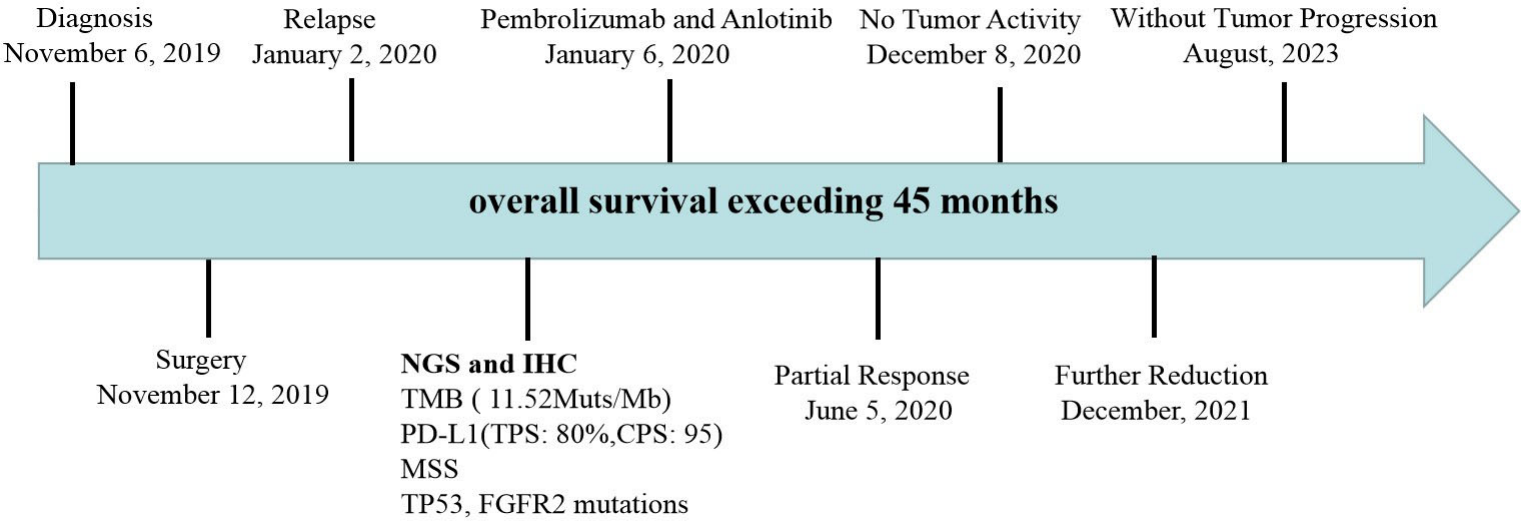
Timeline of treatment process.

After 7 cycles of treatment (June 5, 2020), PET-CT showed that the recurrent malignant tumor in the right lung significantly reduced to 54mm * 43mm. The metabolic activity of the tumor has significantly decreased compared to before, with SUVmax decreasing to 7.4 (**Figures 3C, D**). Tumor biomarker NSE decreases to normal levels (**Supplementary Material 3**). Moreover, the quality of life improved significantly. During this period, the patient experienced adverse drug reactions, including one-degree thyroid-stimulating hormone elevation and two-degree hand-foot syndrome, which was attributed to anlotinib. Therefore, sodium levothyroxine was supplemented 50μg every day and the anlotinib dose was reduced to 8 mg once daily. After 15 cycles therapy (December 8, 2020), PET-CT depicted reduced right anterior metastasis (46 mm * 33 mm) and minimal metabolic activity with SUVmax 2.4 (**Figures 3E, F**). The Response Evaluation Criteria in Solid Tumors (RECIST) version 1.1 evaluation was PR. Even though the tumor had not completely disappeared, there is essentially no tumor activity. Afterwards, pembrolizumab combined with anlotinib continued treatment until December 2021, with further reduction of lung malignancies (**Figures 3G, H**). A total of 34 cycles were used and no adverse reactions of 3-4 degrees were observed. This finding suggests that anlotinib is well tolerated. Currently, the patient is still alive without tumor progression and has overall survival exceeding 45 months until the submission date. The patient is in long-term survival with tumors (**Figure 4**).

## Discussion

PSC occurs predominantly in men with a median age of 68, smoking is the major risk factor. PSC has no specific clinical presentation with a median tumor size of 5 cm (5). Peripheral tumors are more frequent than central tumors, and lesions are primarily localized to the upper lobe (20), which may invade the pleura and ribs, causing pain. However, this study reported an elderly, 73-year-old female patient, non-smoker, presenting with a lesion in the upper lobe of the right lung, who was diagnosed advanced malignant tumors.

The paper showed that the patient developed disease recurrence and metastasis seven weeks following surgery without postoperative adjuvant chemoradiation, confirming that PSC was aggressive, highly malignant, and prone to metastasis. The treatment regimen of PSC is similar to that of NSCLC. Complete surgical resection is currently considered the best treatment for PSC, with a median postoperative survival of eight months (21). Adjuvant chemotherapy in PSC patients is still controversial. A meta-analysis showed that OS in patients receiving adjuvant chemotherapy was significantly longer than those treated with surgery alone, and patients with PSC may benefit from adjuvant chemotherapy (22). Additional studies suggested that adjuvant chemotherapy improves five-year OS for stage II and III disease but not for stage I disease (23). The prognosis of patients with pleomorphic carcinoma is poor, despite surgery and adjuvant chemotherapy, because of early disease relapse. Federico Raveglia et al. (24) studied 20 cases of pleomorphic pulmonary carcinoma and found the median duration of disease-free survival was five months, and the median duration of OS was eight months. Filippo Lococo et al (6) reports that 81% of patients who undergo surgical treatment often experience distant recurrence, even in 62% of stage I tumors undergoing R0 resection. In addition, Ting Gong et al. (25)reported a clinical analysis of 78 cases of surgical treatment for pulmonary sarcomatoid carcinoma, and neither adjuvant chemotherapy nor radiotherapy provided survival advantages.

PD-L1 expression is the only FDA-approved biomarker for ICIs in patients with lung adenocarcinoma (LUAD). In advanced NSCLC patients with high PD-L1 expression (at least 50% of tumor cells), pembrolizumab was associated with significantly longer PFS, OS and fewer adverse events than platinum-based chemotherapy (26, 27). ICIs have improved cancer prognosis but have not been evaluated specifically in advanced PSC.

Studies showed that PD-L1 positive expression was found in 72.3% of surgically removed lung sarcomatoid carcinomas (28). Given the rarity of PSC, prospective studies of the efficacy and toxicity of ICIs in PSC are seldom. Retrospective studies showed a longer PFS and OS with ICIs monotherapy in advanced PSC with PD-L1 high expression (29). Charlotte Domblides et al. (2) found that the advanced PSC patients, regardless of PD-L1 status and second- or further-line accepted nivolumab immunotherapy, had ORR of 40.5% and DCR of 64.8% and median OS of 12.7 months. There was a trend toward higher PD-L1 expression in responsive disease. A retrospective study showed that the median PFS of PD-L1 positive and negative patients was 17.50 months and 6.07 months (30). High expression of PD-L1 provides a biological basis for immunotherapy in PSC patients.

TMB is also an independent predictor of ICIs response, having broad clinical utility regardless of tumor type, PD-L1 expression, or MSI status (31). High TMB (10 variants/Mb) improved NSCLC response and prolonged PFS regardless of PD-LI expression (32). ORR was observed in 29% of patients in the TMB-high group and 6% in the non-TMB-high group (33). The median OS of high TMB patients was 18 months, while the low TMB population was only 1.84 months (2). TMB could be a novel and valuable predictive biomarker of pembrolizumab monotherapy response in recurrent or metastatic advanced solid tumors. More than 60% of PSC patients had MSI-H, PD-L1-positive, or high-TMB (11). The median TMB in PSC was 8.1 mutations/Mb (9), and about 37.9–87.5% of Chinese PSC patients had high TMB, which may benefit from ICIs (11, 34).

Pembrolizumab is a humanized monoclonal anti-PD1 antibody approved by the US FDA for treating solid tumors with high TMB (TMB-high, 10 variants/Mb) and PD-L1 expression and has been widely studied in many malignancies. In KEYNOTE-024, for driver gene-negative advanced NSCLC with high PD-L1 expression, the median OS with pembrolizumab monotherapy and chemotherapy was 26.3 and 13.4 months, respectively (35). There were few studies about pembrolizumab in treating PSC. Our reported patient had high TMB (TMB-high, 11.5variants/Mb) and PD-L1 expression (TPS 80% and CPS 95), and pembrolizumab was selected as an inhibitor of the immune monitoring point, attaining a longer effective response. TMB and PD-L1 expression may be useful potential biomarkers for PSC for predicting the response to pembrolizumab treatment in advanced PSC.

This report presented a simultaneous TP53 mutation in advanced PSC patients. Targeted therapy is a landmark success in NSCLC treatment, with investigators exploring the molecular typing and biological characteristics of PSC. The TP53 gene is the most frequently mutated gene in cancer (11). Strong positive or overexpression of the TP53 gene are poor prognostic factors. Moreover, 45% of patients with pure PSC harbored at least one actionable alteration, mainly including TP53 (74%), KRAS (24%) mutations (11), and EGFR mutations can be detected in a small number of PSC patients (34). Some studies have suggested that tumor angiogenesis may lead to mutations in multiple genes, such as NF1 and TP53 (36). In addition,TP53 mutations were positively associated with specific immune cells and an inflamed immunotype (37), mutations in KRAS, TP53, and MET in LUAD were significantly associated with high expression of PD-L1, the ORR of LUAD patients with TP53 mutation was 39.8% in high expression of PD-L1 group than 18.3% in low expression of PD-L1 (38). Charlotte Domblides reported that high TMB of PSC patients had a TP53 mutation, patients who accepted the ICIs exhibited a trend toward higher survival than TMB low expression (18 months vs. 1.84 months) (2). Genetic mutations may significantly impact the immune microenvironment of lung cancer, but TP53 and KRAS have demonstrated predictive value for ICIs (39). The TP53, STK 11, and EGFR mutations could predict the anti-PD-1 response in LUAD (40).

Angiogenesis plays a crucial role in tumor growth and metastasis, in cancer therapy, blocking this pathway has become a new area of research. Anlotinib is a novel oral multi-target tyrosine kinase inhibitor with broad-spectrum inhibition of tumor angiogenesis and growth, and the main targets include VEGFR2/3, FGFR1-4, PDGFR α/β, c-Kit and Ret (41–43). ALTER 0303 study confirmed that anlotinib improved OS and PFS in third-line or further-line treatment in advanced NSCLC (44), it was approved in May 2018 as a third-line treatment for refractory advanced NSCLC (45).

Antiangiogenic targeted therapy combined with ICIs can produce synergistic effects. The lung cancer mouse model shows that anlotinib increases the infiltration of natural killer cells (NK) and antigen presenting cells (APC), and when combination with ICI, anlotinib has significant synergistic therapeutic benefits (46). Anti-angiogenic drugs improve the tumor microenvironment by resisting tumor angiogenesis, while anti-PD1 immunotherapy can activate immune cells and promote vascular normalization, forming positive feedback circulatory mechanism. Therefore, anti-angiogenic therapy combined with immunotherapy can synergize with tumor cells to improve efficacy (47). The median PFS of PSC who received systemic ICI therapy was 9.6 months (48). Qian et al. (49) retrospectively analysis of 21 PSC patients with the first-line ICIs treatment,the median PFS(mPFS) was 8.0, 9.4, and 9.6 months for immunotherapy alone, immunotherapy combined with anlotinib, and chemoimmunotherapy. In the present study, the patient was received the anlotinib combined with pembrolizumab and had a long time stable disease and a good quality. Therefore, pembrolizumab and anlotinib could represent a promising chemotherapy-free option for treatment-naive patients with advanced PSC.

Adverse drug reactions expressed by the patients in this report included: one-degree thyroid-stimulating hormone elevation and two-degree hand-foot syndrome, which was attributed to anlotinib. No 3-4 degree adverse reactions observed. Considering poor patient tolerance, start using 10mg of anlotinib instead of 12mg. The most common adverse events in the anlotinib group were hypertension, fatigue, thyroid-stimulating hormone elevation, anorexia, hypertriglyceridemia, hand-foot syndrome, and hypercholesterolemia in ALTER 0303 study (44). The major reasons for dose reduction were hand-foot syndrome and hypertension (44). In our case, pembrolizumab combined with anlotinib did increase the incidence of adverse reactions. However, reducing the dosage of anlotinib remains effective. Therefore, it is necessary to consider adverse reactions and clinical efficacy, as future clinical trials are needed to verify the optimal dosage of anlotinib. Pembrolizumab and anlotinib was continued for two years as a maintenance treatment. There is no consensus on how long pembrolizumab maintenance treatment should be continued, and clinical studies generally recommend two years. From follow-up till now, the patient had a good quality of life and continued PR, with survival over 45months. We will continue to follow up on the patients. As far as we know, this is the first report demonstrating the combination of pembrolizumab with anlotinib for advanced PSC with TMB-H, high PD-L1 expression and TP53 mutation. Immunotherapy combined with anti-angiogenic drugs might be the potential and promising strategy for treating PSC. However, its effectiveness and safety need to be verified in the future.

## Data availability statement

The raw data supporting the conclusions of this article will be made available by the authors, without undue reservation.

## Ethics statement

The studies involving humans were approved by Ganzhou Hospital-Nanfang Hospital,Southern Medical University. The studies were conducted in accordance with the local legislation and institutional requirements. The participants provided their written informed consent to participate in this study. Written informed consent was obtained from the individual(s) for the publication of any potentially identifiable images or data included in this article.

## Author contributions

SW: Writing – original draft, Project administration. ShaW: Data curation, Writing – review & editing. XL: Writing – review & editing, Software. CZ: Writing – review & editing, Methodology. FQ: Writing – review & editing. CW: Writing – original draft, Validation. WZ: Writing – review & editing, Validation.

## Supplementary material

Supplementary Material 1 Pre-treatment PET-CT results. **(A, B)**, 18F-FDG PET/CT shows a soft tissue mass near the hilum of the right upper lobe of the lung (solid tumor, dmax = 62 mm * 49 mm), FDG metabolism is increased, and SUVmax 32. **(C, D)**, enlarged lymph nodes in the mediastinum.

Supplementary Material 2 The results of NGS and IHC analysis.

Supplementary Material 3 The trend of changes in NSE(ng/ml) in serum (normal levels, <16.3 ng/ml).
