# Supplementary figures and images for: Pembrolizumab combined with anlotinib improves therapeutic efficacy in pulmonary sarcomatoid carcinoma with TMB-H and PD-L1 expression: a case report and literature review
Source: Front Immunol. 2023 Oct 23;14:1274937. doi: 10.3389/fimmu.2023.1274937 (PMC10626500; doi:10.3389/fimmu.2023.1274937)

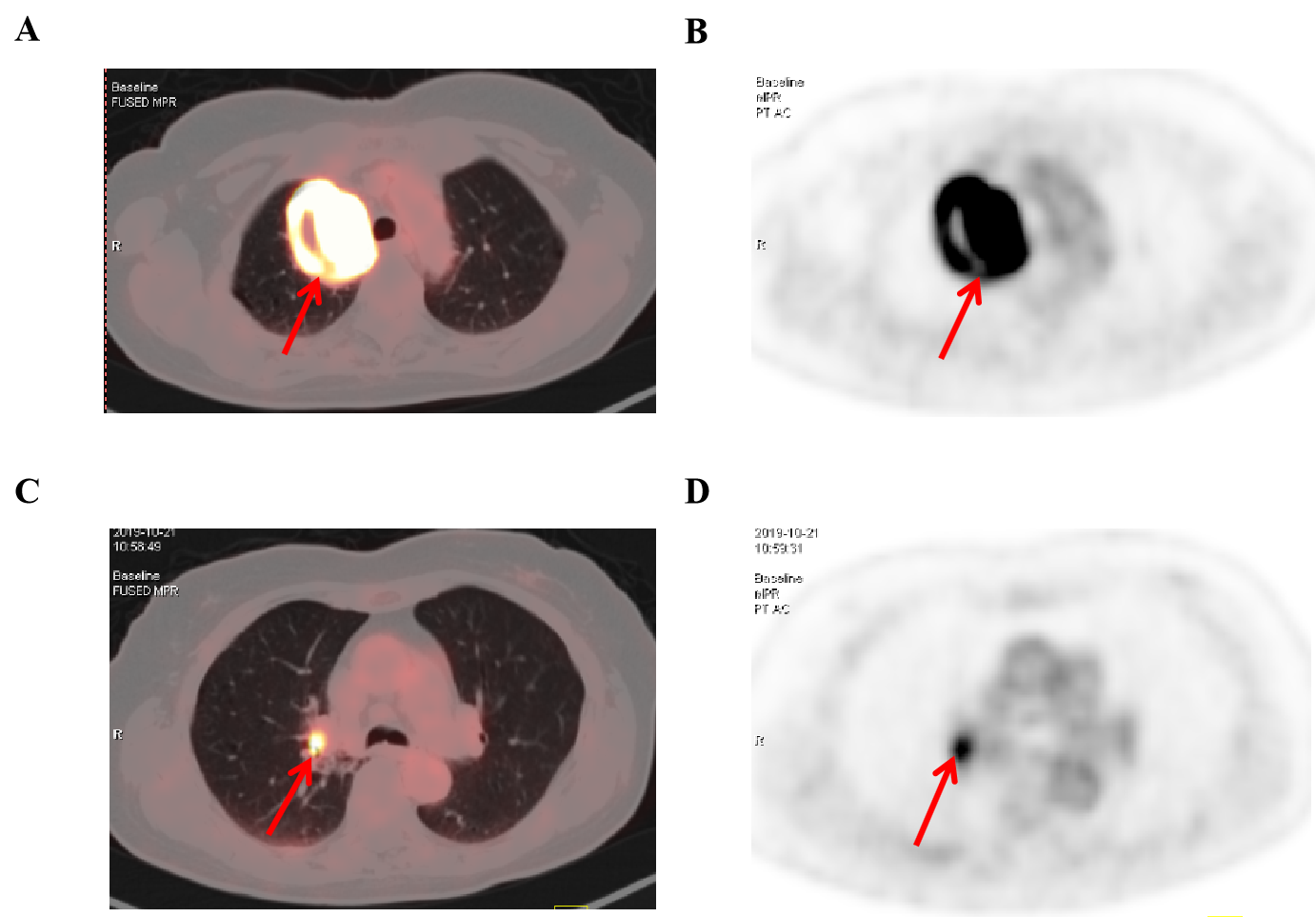

Supplement: Supplementary Material 1 — Pre-treatment PET-CT results. (A, B), 18F-FDG PET/CT shows a soft tissue mass near the hilum of the right upper lobe of the lung (solid tumor, dmax = 62 mm * 49 mm), FDG metabolism is increased, and SUVmax 32. (C, D), enlarged lymph nodes in the mediastinum. [file Image_1.png]

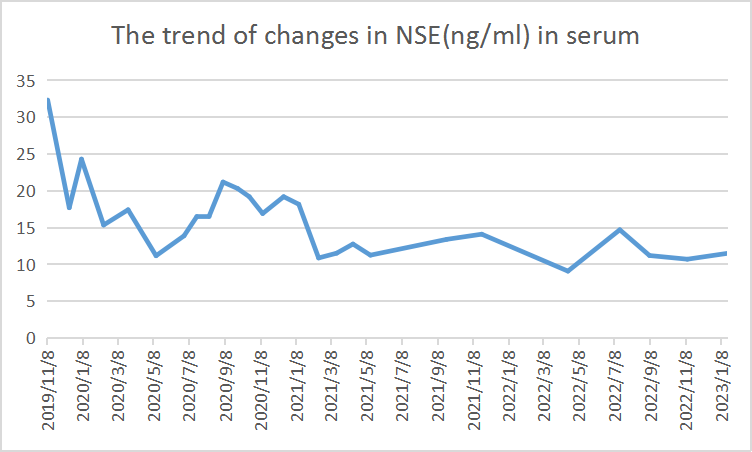

Supplement: Supplementary Material 2 — The results of NGS and IHC analysis. [file Image_2.png]
