# Supplementary material for: Pembrolizumab combined with anlotinib improves therapeutic efficacy in pulmonary sarcomatoid carcinoma with TMB-H and PD-L1 expression: a case report and literature review
Source: Front Immunol. 2023 Oct 23;14:1274937. doi: 10.3389/fimmu.2023.1274937 (PMC10626500; doi:10.3389/fimmu.2023.1274937)
Supplement: Supplementary Material 3 — The trend of changes in NSE(ng/ml) in serum (normal levels, <16.3 ng/ml). [file Table_1.docx]

**Table 1** The results of next-generation sequencing (NGS) analysis

| **Cohort** | **Value** | |
| --- | --- | --- |
| Microsatellite | Microsatellite stable | |
| Tumor mutation burden | 11.52 Mutants/Mb | |
| PD-L1 expression | tumor proportion score [TPS]^a^: 80%  combined positive score [CPS]^b^: 95 | |
| Gene | Mutation | abundance/  mutation frequency. |
| ARID2 | p.L1321Qfs*10 Exon15 | 40.0% |
| APC | p.T1267S Exon16 | 26.6% |
| TP53 | p.p47Rfs*76 Exon4 | 25.3% |
| PTPRD | p.G251Efs*17 Exon20 | 24.4% |
| FGFR2 | p.p443L Exon10 | 24.1% |
| RB1 | p.Q471E Exon15 | 24% |
| ARID1A | p.S22L Exon1 | 23.9% |
| NF1 | p.Q369* Exon10 | 23.9% |
| TERT | . . | 17.9% |
| BCOR | p.D208v Exon4 | 15.8% |
| SRC | p.R271L Exon9 | 13.5% |
| MLL3 | p.E1097K Exon20 | 11.5% |
| IGLL5 | p.F141V Exon3 | 10.8% |
| NUP93 | p.R175* Exon6 | 2.6% |

^a^TPS was defined as the percentage of viable tumor cells with partial or complete membrane staining of PD-L1 in at least 100 viable tumor cells.

^b^CPS was defined as the number of PD-L1 stained cells (tumor cells, lymphocytes, macrophages) divided by the number of all tumor cells and multiplied by 100.
